# Synthetic Long Peptide Influenza Vaccine Containing Conserved T and B Cell Epitopes Reduces Viral Load in Lungs of Mice and Ferrets

**DOI:** 10.1371/journal.pone.0127969

**Published:** 2015-06-05

**Authors:** S. K. Rosendahl Huber, M. G. M. Camps, R. H. J. Jacobi, J. Mouthaan, H. van Dijken, J. van Beek, F. Ossendorp, J. de Jonge

**Affiliations:** 1 Centre for Infectious Disease Control (Cib), National Institute for Public Health and the Environment (RIVM), Bilthoven, the Netherlands; 2 Department of Immunohematology and Blood Transfusion, Leiden University Medical Center (LUMC), Leiden, the Netherlands; Georgia State University, UNITED STATES

## Abstract

Currently licensed influenza vaccines mainly induce antibodies against highly variable epitopes. Due to antigenic drift, protection is subtype or strain-specific and regular vaccine updates are required. In case of antigenic shifts, which have caused several pandemics in the past, completely new vaccines need to be developed. We set out to develop a vaccine that provides protection against a broad range of influenza viruses. Therefore, highly conserved parts of the influenza A virus (IAV) were selected of which we constructed antibody and T cell inducing peptide-based vaccines. The B epitope vaccine consists of the highly conserved HA2 fusion peptide and M2e peptide coupled to a CD4 helper epitope. The T epitope vaccine comprises 25 overlapping synthetic long peptides of 26-34 amino acids, thereby avoiding restriction for a certain MHC haplotype. These peptides are derived from nucleoprotein (NP), polymerase basic protein 1 (PB1) and matrix protein 1 (M1). C57BL/6 mice, BALB/c mice, and ferrets were vaccinated with the B epitopes, 25 SLP or a combination of both. Vaccine-specific antibodies were detected in sera of mice and ferrets and vaccine-specific cellular responses were measured in mice. Following challenge, both mice and ferrets showed a reduction of virus titers in the lungs in response to vaccination. Summarizing, a peptide-based vaccine directed against conserved parts of influenza virus containing B and T cell epitopes shows promising results for further development. Such a vaccine may reduce disease burden and virus transmission during pandemic outbreaks.

## Introduction

In influenza infection, both antibodies and T cell responses play an important role in viral clearance and protection against disease. In general, antibodies bind to the virus particles to prevent infection or spread of the virus, while T cells can kill virus-infected cells and provide help to other cells of the immune system. Many of the traditional influenza vaccines are only aimed at the induction of antibodies and are often poor inducers of T cell responses [1, 2]. Since these vaccines mainly confer protection via antibodies directed against the highly variable surface proteins hemagglutinin (HA) and neuraminidase (NA), protection is subtype or strain-specific. Due to mutations in the antigenic site, so-called antigenic drift, influenza virus can escape vaccine-induced immunity and consequently regular vaccine updates are required. In addition, current vaccines do not provide protection against newly emerging influenza subtypes, which has multiple times led to the outbreak of a pandemic. Universal influenza vaccines could provide a solution to these issues, and are therefore an important subject in influenza vaccine research.

A universal vaccine should target conserved parts of the virus, thereby inducing cross-protection against multiple influenza subtypes. The globular head of HA is highly variable, while the N-terminal fusion peptide of the HA2 subunit is highly conserved. The fusion protein plays a crucial role in the fusion process of the viral envelope with the host membrane. Therefore, antibodies directed against the highly conserved fusion protein might be able to neutralize infectivity of influenza virus by inhibiting fusion. Chun *et al* showed that it is indeed possible to induce antibodies that specifically bind to the fusion peptide, making it a promising target for vaccine development [3–5]. Another highly conserved region is the extracellular domain of the M2 protein (M2e) [6]. Antibodies directed against the M2e domain are not as immunogenic as antibodies directed against the globular head of HA or NA, since M2e is not directly accessible to antibodies. However, M2e-specific antibodies can limit spread of the virus by binding virus particles to the cell, thereby preventing release into the extracellular fluid [7]. Furthermore, these antibodies have been shown to play a role in antibody-mediated cytotoxicity [8].

The internal proteins of influenza virus also contain several conserved regions, as reviewed by Stanekova *et al*. Screening of PBMCs from healthy donors showed that NP is a major target of immunodominant cytotoxic T cell (CTL) responses [9–11]. Other important targets of T cell responses to influenza are located in matrix protein 1 (M1) and polymerase basic protein 1 (PB1) [12]. Recently, two studies have shown the importance of T cell mediated protection in subjects lacking strain specific pre-existing humoral immunity. Sridhar *et al*. described that individuals with higher numbers of preexisting CD8^+^ T cells specific for conserved epitopes developed less severe illness after infection with pandemic H1N1 influenza virus [13]. Wilkinson *et al*. monitored T cell responses of healthy volunteers following influenza challenge, and observed that lower virus shedding and less severe illness correlated with the presence of influenza-specific cytotoxic CD4^+^ T cells [14].

Another promising strategy for universal vaccine development is therefore to induce T cell responses to conserved parts of the virus, for example by vaccination with peptides. The first and most successful peptide-based vaccine for virus infections is a therapeutic vaccine against human papilloma virus (HPV). This vaccine contains synthetic peptides of at least 25 amino acids long, directed against viral oncoproteins and induced vaccine specific CD4^+^ and CD8^+^ T cell responses in all patients [15]. While peptides of 8–10 amino acids long can bind directly to MHC class I, long peptides require processing by professional APCs, which reduces the chance of inducing tolerance by peptide vaccination [16]. Furthermore, long peptides often contain multiple epitopes that are not restricted to a certain MHC haplotype, thereby broadening the potential response at both the individual and population level [17]. Another advantage is that, in addition to CD8 epitopes, these longer peptides often contain CD4 epitopes. Specific CD4^+^ T helper cells support effective co-stimulation during priming of CD8^+^ T cells and promote memory CD8^+^ T cells [18–21]. Depending on the peptides that are selected, this strategy can be applied for a wide range of viral infections.

We aimed to develop a vaccine capable of providing cross-protection against multiple influenza A subtypes, by inducing both T cell and antibody responses directed towards conserved parts of the virus. Therefore, 25 overlapping long synthetic peptides were selected based on conservation in different subtypes of influenza viruses. Furthermore, peptides containing the highly conserved HA2 fusion peptide and M2e epitope were coupled to a CD4 helper epitope to increase their immunogenicity. Mice and ferrets were vaccinated with these peptide vaccines, either alone or in combination, to evaluate immunogenicity of both the antibody and T cell components. The protective capacity of these promising vaccine candidates was evaluated in influenza A challenge models in C57BL/6, BALB/c mice and ferrets. The vaccine was shown to be capable of reducing viral load in the lungs.

## Materials and Methods

### Ethics Statement

This study was approved by the Committee on Animal Experimentation of the Netherlands Vaccine Institute (Bilthoven, the Netherlands) under permit numbers 201100334, 201200164 and 201200281. Animal handling was carried out in accordance with relevant Dutch national legislation, including the 1997 Dutch Act on Animal Experimentation. When possible, mice were anesthesized by isoflorane in O_2_ to minimize suffering. For all challenge experiments human endpoints were set prior to the start of the study to prevent unnecessary suffering of the animals. Endpoints were based on clinical signs of influenza disease such as heavy breathing, activity, posture and fur condition and on decrease in bodyweight compared to weight prior to challenge. If animals reached these endpoints they were euthanized.

### Selection of 25 Synthetic Long Peptide (25 SLP) Vaccine

H1N1 and H5N1 viruses, with a collection date from 1970 until 2010, were selected from the NCBI Influenza Virus Sequence Database. All hosts and countries/regions were included, only full-length sequences were included, and lab strains were excluded. Using the alignment tool, conserved regions were identified for each protein of the influenza virus. Then, influenza proteins NP, PB1 and M1 were selected since these contained the largest amount of conserved regions which also coded for a number of known CD8 and CD4 epitopes (S1 Table) [12, 22–24]. From these regions, 25 overlapping peptides were selected: eleven from NP, eight from PB1 and six from M1. Final length of these peptides was based on the inclusion of known epitopes as described in S1 Table, therefore the length of these peptides ranges from 26–34 amino acids (Table 1).

**Table 1:** Conservancy analysis peptides.

Peptide	Sequence	H1N1	H3N2	H2N2	H5N1	H7N9	HK-X31
NP_17-46_	GERQNATEIRASVGRMIGGIGRFYIQMCTE	96.7	90	93.3	96.2	93.3	96.7
NP_37-65_	GRFYIQMCTELKLSDYEGRLIQNSLTIER	93.1	93.1	100	96.6	93.1	100
NP_56-81_	LIQNSLTIERMVLSAFDERRNKYLEE	96.2	96.2	100	92.3	92.3	100
NP_67-93_	VLSAFDERRNKYLEEHPSAGKDPKKTG	100	100	100	96.3	96.3	100
NP_191-220_	ELIRMIKRGINDRNFWRGENGRKTRIAYER	93.3	93.3	100	96.7	96.7	96.7
NP_212-244_	GRKTRIAYERMCNILKGKFQTAAQKAMMDQVRE	90.9	90.9	97	93.9	93.9	100
NP_234-263_	AQKAMMDQVRESRNPGNAEFEDLTFLARSA	90	86.7	90	90	90	100
NP_254-281_	EDLTFLARSALILRGSVAHKSCLPACVY	96.4	92.9	96.4	96.4	96.4	100
NP_317-349_	RPNENPAHKSQLVWMACHSAAFEDLRVSSFIRG	100	93.9	97	100	100	90.9
NP_378-403_	TLELRSRYWAIRTRSGGNTNQQRASA	96.2	96.2	100	100	100	100
NP_394-420_	GNTNQQRASAGQISIQPTFSVQRNLPF	92.6	92.6	96.3	100	92.6	100
PB1_1-41_	MDVNPTLLFLKVPAQNAISTTFPYTGDPPYS	96.8	100	100	96.4	96.8	100
PB1_21-51_	TFPYTGDPPYSHGTGTGYTMDTVNRTHQYSE	100	100	100	100	96.8	100
PB1_401-434_	ASLSPGMMMGMFNMLSTVLGVSILNLGQKRYTKT	97.1	97.1	94.1	100	97.1	100
PB1_474-503_	GINMSKKKSYINRTGTFEFTSFFYRYGFVA	96.7	96.7	100	100	100	100
PB1_494-523_	SFFYRYGFVANFSMELPSFGVSGINESADM	96.7	100	100	100	100	100
PB1_534-562_	MINNDLGPATAQMALQLFIKDYRYTYRCH	100	100	100	100	100	100
PB1_588-617_	GLLVSDGGPNLYNIRNLHIPEVCLKWELMD	100	100	100	100	100	100
PB1_699-727_	FPSSSYRRPVGISSMVEAMVSRARIDAR	100	96.4	100	100	100	100
M1_31-60_	VFAGKNTDLEALMEWLKTRPILSPLTKGIL	100	100	100	96.7	93.3	96.7
M1_51-80_	ILSPLTKGILGFVFTLTVPSERGLQRRRFV	100	100	100	96.7	100	
M1_69-98_	PSERGLQRRRFVQNALNGNGDPNNMDKAVK	96.7	100	96.7	96.7	100	100
M1_87-115_	NGDPNNMDKAVKLYRKLKREITFHGAKEI	89.7	100	96.6	89.7	89.7	100
M1_167-194_	TTTNPLIRHENRMVLASTTAKAMEQMAG	100	92.9	100	96.4	100	100
M1_180-206_	VLASTTAKAMEQMAGSSEQAAEAMEVA	100	96.3	100	100	100	100
M2e	SLLTEVETPIRNEWGSRSNDSSD	73.9	91.3	91.3	76.2	56.5	87
HA2	GLFGAIAGFIENGWEG	87.5	93.8	87.5	87.5	100	93.8

Numbers depict the percentage of conservancy.

### Conservancy Analysis

Conservancy was analyzed using LALIGN from the Swiss Institute for Bioinformatics, based on a paper by Huang and Miller [25]. Peptides used in the vaccines were aligned with proteins of vaccine strains and possible pandemic strains from different influenza A subtypes: A/California/07/2009 (H1N1), A/Victoria/361/2011 (H3N2), A/Japan/305/1957 (H2N2), high pathogenicity avian influenza virus (HPAI) A/turkey/Turkey/1/2005 (H5N1), A/Anhui/1/2013 (H7N9) and HK-X31 (a mouse adapted influenza strain containing the HA and NA proteins of A/Aichi/68 and internal genes from A/Puerto Rico/8/34). Sequences of A/Anhui/1/2013 was obtained from GISAID and provided by Lei Yang from the WHO Chinese National Influenza Center (Beijing, China).

### Peptide Synthesis

All peptides used in these experiments were prepared by normal Fmoc-chemistry using preloaded Tentagel resins (PyBop/NMM) for in situ activation, and 20% piperidine in NMP for Fmoc removal. Couplings were performed for 60 minutes with 6-fold acylating species. After final Fmoc removal, peptides were cleaved with TFA/H_2_O 19/1 (v/v) containing additional scavengers when C (triethylsilane) or W (ethanethiol) were present in the peptide sequence. Peptides were isolated by ether/pentane 1:1 (v/v) precipitation and the product was isolated by centrifugation. Following air-drying at 40°C, peptides were dissolved in acetic acid/water 1:10 (v/v) and lyophilized. Purity of the peptides was analyzed using UPLC-MS (Acquity, Waters) and integrity was confirmed by using Maldi-Tof mass spectrometry (Microflex, Bruker) [26]. All peptides used in these experiments were synthesized at the LUMC (Leiden, the Netherlands).

### Vaccines

The B epitopes vaccine for mice contained the following long peptides; SLLTEVETPIRNEWGSRSNDSSD deduced from M2e, and GLFGAIAGFIENGWEG deduced from the HA2 fusion peptide. For the ferret experiments, the M2e peptide and the HA2 fusion peptide were adapted for the Influenza A/turkey/Turkey/1/2005 H5N1 virus, resulting in the following sequences: SLLTEVETPTRNEWESRSSDSSD and GLFGAIAGFIEGGWQG, respectively. Adaptations are shown in bold. The HA2 and M2e peptides were coupled N-terminally to the CD4 helper peptide CPKYVKQNTLKLATG (HA_321-335_) for murine experiments or to the universal helper peptide PADRE (UKXVAAWTLKAAU; U = d-Ala and X = cyclohexylalanine) for ferret experiments [27]. Therefore, when there is referred to the B epitopes as a vaccine, these are always conjugated to one of the helper peptides. The 25 SLP vaccine contained 25 synthetic long overlapping peptides as shown in Table 1. Mice received 20 nmol of each of the indicated peptides admixed in PBS, Incomplete Freund’s Adjuvant (IFA, Invivogen) and 5 nmol Pam3CysSK4 (Invivogen). C57BL/6 mice also received a 2 SLP vaccine containing HA_321-335_ and RGVQIASNENMETMESSTLE (NP_361-380_) alone or in combination with the B epitopes. Ferrets received a combination of the 25 SLP and B epitopes vaccine, with 200 μg of each peptide, admixed with 10 nmol Pam3CysSK4 either with or without IFA, as indicated. Mice received their vaccination in a volume of 0.1 mL, and ferrets in 0.5 mL. Mice and ferrets in the positive control groups were vaccinated by means of intranasal infection (i.n.) with a low dose of live HK-X31 virus (1*10^2^ (first infection) or 1*10^3^ (second infection) TCID_50_) or Highly Pathogenic Avian Influenza virus (HPAI) H5N1 influenza A/turkey/Turkey/1/2005 virus (1*10^4^ TCID_50_), respectively. Negative control animals received the respective adjuvant mixture without peptides.

### Immunization and Challenge Experiments in Mice

Female C57BL/6 and BALB/c mice were obtained from Jackson Laboratory and used at 8–10 weeks of age. Mice were vaccinated subcutaneously (s.c.) on days 0 and 14, with respective vaccines, and prior to primary vaccination serum samples were collected. I.n. vaccinations were performed under anesthesia with isoflurane in O_2_. On day 28, serum samples were collected and mice were challenged i.n. with 50 μl of a sub-lethal dose of 1*10^5^ pfu HK-X31 in PBS. Starting from challenge, bodyweight and clinical signs were recorded daily. Five days after challenge, four mice (C57BL/6) or six mice (BALB/c) per group were sacrificed by bleeding and cervical dislocation under anesthesia with isoflurane in O_2_. Spleens and lymph nodes (LNs) were processed directly for use in T cell assays, and lungs were excised and stored at -80°C for further analysis. The remaining mice, six per group for C57BL/6 and ten for BALB/c, were sacrificed two weeks after challenge. None of the mice needed to be euthanized prior to the experimental endpoint.

### Immunization and Challenge Experiments in Ferrets

Female ferrets (*Mustela putorius furo*), aged 6–12 months, were obtained from Triple F Farms. All ferrets were confirmed to be negative for previous circulating influenza virus infection and Aleutian disease. A temperature transponder (DST micro T, Star-Oddi) was implanted in the peritoneal cavity 14 days prior to start of the experiment, which recorded the temperature every 30 minutes. Area under curve (AUC) above the baseline temperature was calculated for each group from day of viral challenge up to their sacrifice, to analyze fever development. On days 0 and 21, ferrets were vaccinated with respective vaccines. Two weeks after booster vaccination, they were challenged with 1*10^5^ TCID_50_ H5N1 Influenza A/turkey/Turkey/1/2005 in 3 mL via the intratracheal route (i.t.). We decided to use the i.t. route, because vaccine-induced T cells reside mainly in the lungs and less in the nasal cavity, which is the target of i.n. challenge. Moreover, this model induces disease similar to that seen in humans after H5N1 infection [28, 29]. Prior to challenge, ferrets were moved to BSL-3 isolators and from this point on, each day clinical signs were scored and every other day ferrets were weighed. Ferrets exhibiting pre-determined endpoints were euthanized by cardiac bleeding under anesthesia with ketamine (5 mg/kg) and medetomidine (0.1 mg/kg). Five days after challenge, ferrets were sacrificed and distal sections of the right lung and accessory lobes were isolated and stored at < -70°C for further virological analysis. Furthermore, serum was isolated from blood collected at day 35 and stored at -20 until further use. Blood sampling, immunizations and challenge were performed under anesthesia by ketamine (5 mg/kg) and medetomidine (0.1 mg/kg). After handling, the anesthesia was antagonized with atipamezole (0.25 mg/kg) with the exception of challenged ferrets when anesthesia was not antagonized to avoid a sneezing reflex.

### Peptide ELISA

Streptavidin plates (Euro Diagnostica AB) were coated with HA2 or M2e peptide-biotin in a concentration of 1 nmol/ml PBS for one hour at RT. After washing the plates three times with wash buffer (PBS with 0.05% Tween-20), the plates were blocked with block buffer (5% skim milk powder (Sigma Aldrich) in PBS) for one hour at RT. Subsequently, plates were washed, sera were diluted in dilution buffer (PBS containing 1% BSA (Roche)), in two-step dilutions and incubated for two hours at RT. Next, plates were washed and goat α-mouse IgG, IgG1, IgG2a, IgG2b or IgG2c HRP (Southern Biotech) in a dilution of 1:5000 or goat α-ferret IgG (Acris antibodies) in a dilution of 1:10,000 in dilution buffer were added to the wells. After one hour incubation at RT, 50 μl TMB (Sigma) was added and the reaction was stopped with 50 μl 1M H_2_SO_4_. Absorbance was read at 450 nm.

### Virus ELISA

Immulon II (Thermo Scientific) plates were coated with HK-X31 which had been purified by sucrose gradient centrifugation, in a concentration of 400 ng/ml HA as determined by calculating 1/3 of total protein measured by a Pierce assay. Alternatively, plates were coated with a whole inactivated virus (WIV) prepared from the H5N1 vaccine strain NIBRG-23 in a concentration of 1200 ng/ml HA. Following O/N incubation, plates were washed with wash buffer (water with 5% Tween-80) and sera were diluted in two-fold steps in dilution buffer (PBS with 0.1% Tween-80). Then, plates were incubated at 37°C and after one hour, goat α-mouse IgG HRP conjugate (Southern Biotech) was added in a concentration of 1:5000 or goat α-ferret IgG HRP conjugate in a concentration of 1:10,000 (diluted in PBS with 0.5% Protifar (Nutricia)) and plates were incubated for another hour at 37°C. Next, wells were washed with wash buffer and substrate (NaAC, TMB and H_2_O_2_) was added to the wells. After 10 minutes, 100 μL/well of 2M H_2_SO_4_ was added per well to stop the reaction and OD was measured at 450 nm.

### Enzyme-Linked Immunospot (ELISpot) Assay

IFN-γ ELISpot assays were performed according to the manufacturer’s protocol (U-Cytech). Spleens and inguinal lymph nodes were homogenized and passed through 70 μM filters (BD Bioscience). Then, spleen cells and LN cells were washed with RPMI (containing 10% FCS, Penicillin, Streptomycin and Glutamin) and counted using a Casy cell counter (Roche). Cells were plated in a concentration of 4*10^5^ cells/well in an IFN-γ antibody coated PVDF membrane plate (MSIP plates Milipore). Cells were stimulated in duplo with 1 μg of two NP-derived peptides selected from the 25 overlapping peptides pool (FYIQMLTEL and AYERMCNIL). After O/N incubation, spots were visualized according to the manufacturer’s protocol (U-Cytech).

### Virological Analysis

Lungs were homogenized using FastPrep (MP Biomedicals) homogenizer and clarified by low speed centrifugation. Virus titers were determined by end-point titration on Madin-Darby canine kidney (MDCK) cells. In short, MDCK cells were seeded in 96-well plates at a density of 1–5 × 10^4^ cells/well and incubated at 37°C until 90–100% confluence was reached. The cells were inoculated in quadruplets/sextuplets with 200/100 μL of homogenized lungs and diluted five-fold serially. After six days of incubation at 37°C, wells were scored for cytopathic effects (CPE) and infection was confirmed by an Haemagglutination assay. The TCID_50_ titer was determined by the Reed and Muench method [30].

### Statistical Analysis

All data were log transformed and tested for normality by the d’Agostino-Pearson test. Normally distributed data were analyzed by the unpaired-t test and not normally distributed data were analyzed by the Mann-Whitney test. All statistical analysis was performed using Graph Pad Prism 6.04 (GraphPad Software Inc., San Diego, CA).

## Results

### Design of Universal Long Peptide Influenza Vaccine

For the development of a universal influenza vaccine, we included either T cell or B cell epitopes, derived from conserved parts of influenza A virus. At time of peptide selection, H1N1 had just evolved into a pandemic and H5N1 viruses posed a threat of inducing a new pandemic. Therefore, selection of the long overlapping peptides was based on these two virus subtypes. Table 1 shows sequences of all peptides included in the vaccines and conservancy analysis of these peptides against several subtypes of influenza A viruses. A/California/07/2009 (H1N1) and A/Victoria/361/2011 (H3N2) were included in the 2013–2014 seasonal vaccine. A/Leningrad/137/1957 (H2N2), A/turkey/Turkey/1/2005 (H5N1), and A/Anhui/1/2013 (H7N9) are of subtypes that could possibly cause a new pandemic. As shown in Table 1, HA2 fusion peptide and all T cell peptides are highly conserved in all, over 3000, strains analyzed, as illustrated by a similarity of 85% or higher. These peptides also contain a high number of known CD8 and CD4 T cell epitopes presented by a number of different MHC molecules (S1 Table). M2e protein also has large sequence similarities, although sequence similarity is not as high as for the HA2 fusion peptide. In mice, the peptides were admixed with IFA and Pam3CysSK4 as an adjuvant combination. Although, IFA is not suitable for use in humans, it was included to guarantee an optimal adjuvant effect.

### Immunogenicity of B Epitopes in Mice

**Fig 1:**
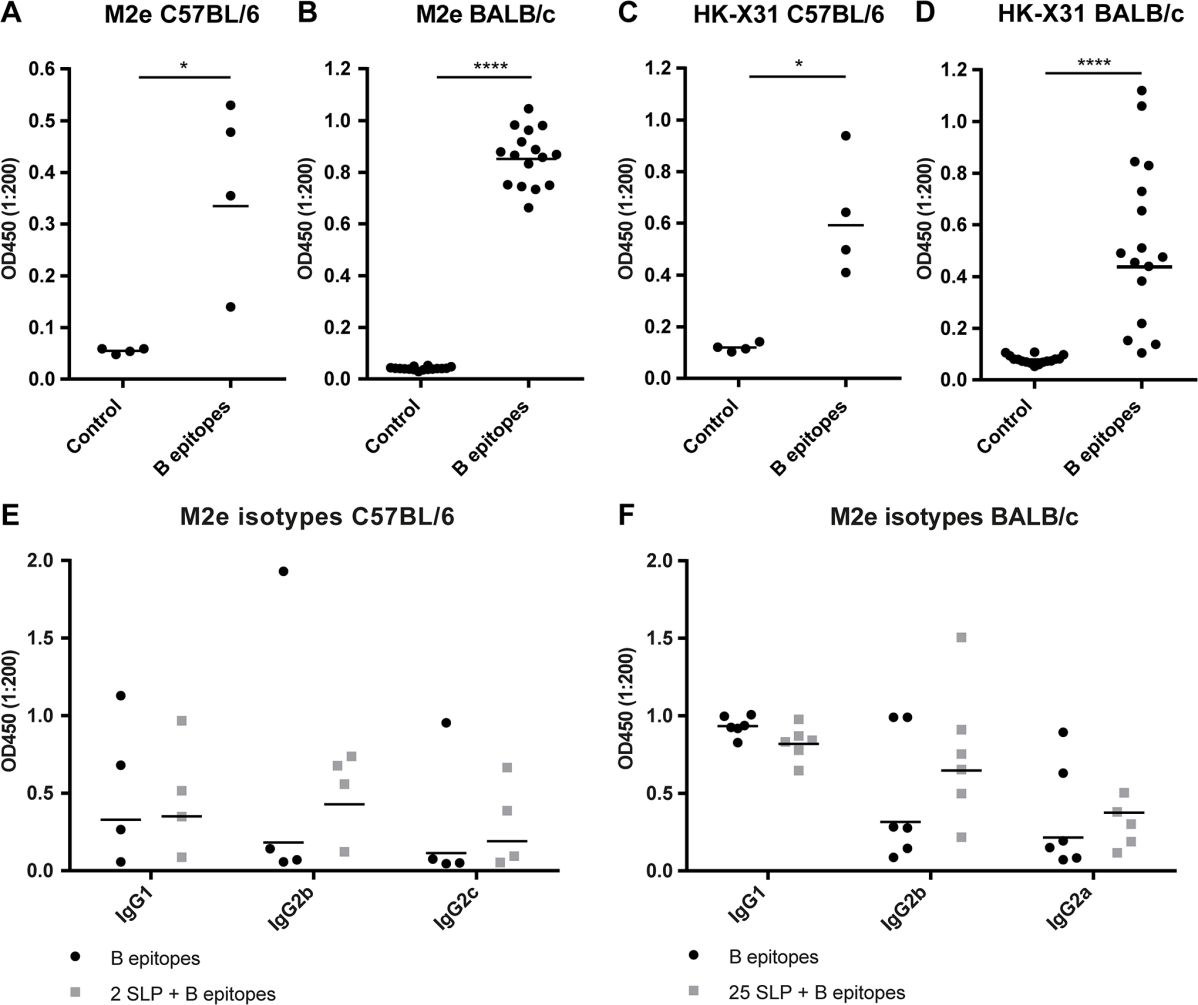
Immunogenicity of B epitopes. Total IgG antibody levels as measured by an ELISA in sera of C57BL/6 and BALB/c mice vaccinated with B epitopes, two weeks after booster vaccination. M2e specific antibodies in C57BL/6 mice **(A)** and BALB/c mice **(B)** and antibodies directed to HK-X31 in C57BL/6 mice **(C)** and BALB/c mice **(D)**. IgG subtyping on M2e antibody responses showed that the addition of T epitopes had no negative effect in C57BL/6 mice **(E)** and BALB/c mice **(F)**. When a Gaussian distribution was found data were analyzed with an unpaired t-test, which was the case for the BALB/c experiments. For C57BL/6 experiments a Mann-Whitney test was performed *p = <0.05, ****p = <0.0001.

Evaluation of the B epitopes vaccine was performed in C57BL/6 and BALB/c mice. Sera collected 14 days post booster vaccination were analyzed by ELISA for HA2-specific and M2e-specific antibody responses, to determine whether immunization with B epitopes induced vaccine-specific antibodies. No HA2 responses were detected in an HA2-specific ELISA (data not shown). M2e-specific IgG antibodies, however, were detected in sera of C57BL/6 and BALB/c mice, proving the immunogenicity of the M2e B cell epitope (Fig 1A and 1B). To evaluate whether these antibodies could recognize the epitopes in their natural conformation, the sera were analyzed by ELISA for recognition of intact influenza virus. Specific antibodies in the serum of both C57BL/6 and BALB/c mice bound to the complete virus particles (Fig 1C and 1D). Therefore, antibodies induced by the vaccine are not only specific for the M2e peptide, but are also capable of recognizing the epitopes in their natural conformation, which is critical for a protective immune response.

### Effect of Addition SLP on Antibody-Response

IgG antibody responses to M2e were further subtyped for IgG1, IgG2b or IgG2a/IgG2c. C57BL/6 mice have a more Th1-skewed response that is correlated with IgG2a/IgG2c antibodies [31]. However, no differences were found between the different subtypes (Fig 1E). BALB/c mice have a more Th2-skewed response that correlates with higher levels of IgG1 antibodies. As expected, BALB/c mice showed a markedly higher IgG1 response (Fig 1F) [32]. These types of antibody response alone are often not sufficient for complete protection against influenza virus infection, therefore we also evaluated the addition of T cell-inducing components to the vaccine. Primary evaluation was performed in C57BL/6 mice. Mice were vaccinated with the B epitopes to which two known T epitopes, HA_321-335_ and NP_361-380_, (2 SLP + B epitopes) were added. HA_321-335_ is the same peptide as used for conjugation to the B epitopes, NP_361-380_ contains a known CD8 peptide that is dominant in the murine response to influenza [33]. Both peptides contain epitopes specific for this H-2^b^ mouse strain. Fig 1E shows that the addition of 2 SLP does not affect the antibody response. Sera of BALB/c mice vaccinated with the B epitopes and 25 SLP (25 SLP + B epitopes), screened for M2e-specific antibodies, showed no differences between the B epitopes alone or the 25 SLP + B epitopes. Thus, even the addition of 25 SLP does not negatively affect antibody responses.

### 25 SLP Induces Specific T Cell Responses

**Fig 2:**
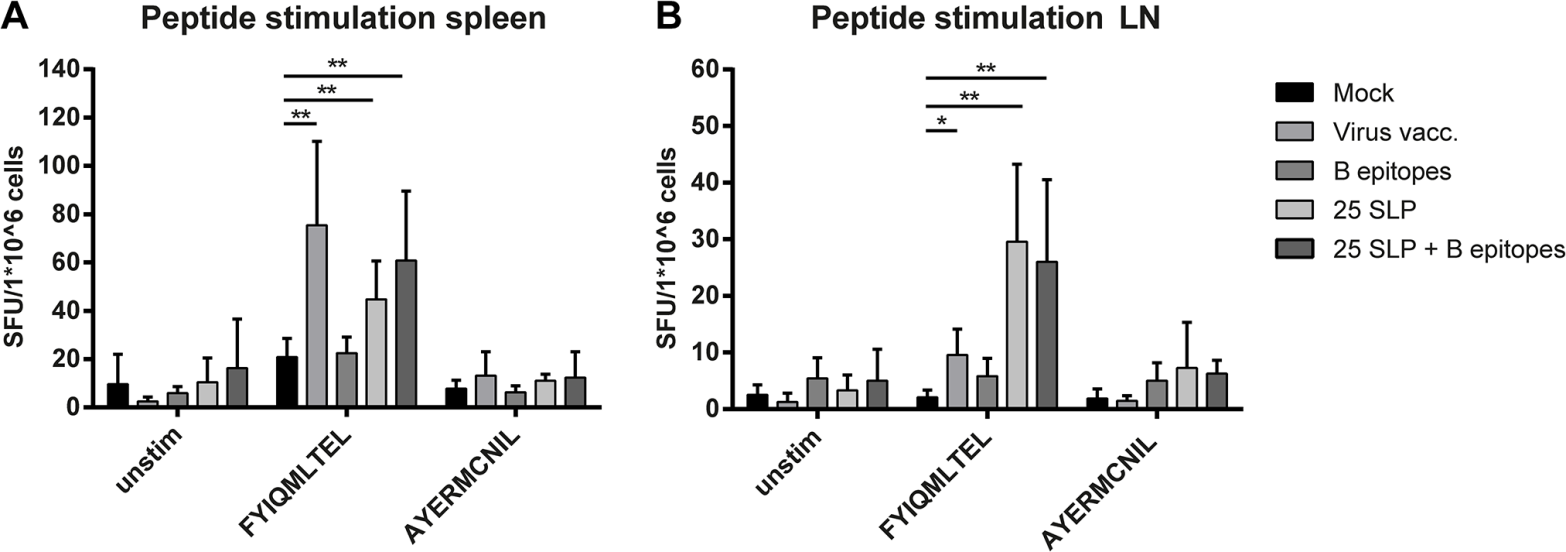
T epitope-specific responses. IFN-γ ELISpot of splenocytes and LN cells of BALB/c mice five days after viral challenge. Results are shown as spot forming units (SFU) per 1*10^6^ cells. Responses in spleen cells **(A)** and LN cells **(B)** after O/N incubation with either medium, FYICMLTEL or AYERMCNIL. Data were log-transformed and analyzed by Mann-Whitney test. *p = <0.05 **p = <0.01.

In BALB/c mice, we analyzed whether the 25 SLP vaccine induced T cell responses in an IFN-γ ELISpot assay, on spleen cells and inguinal LNs isolated five days after viral challenge. Cells were stimulated overnight with two short conserved peptides derived from the NP protein (FYIQMLTEL and AYERMCNIL). These two peptides were selected from 25 SLP based on MHC class I prediction of BALB/c epitopes by the ANN method of the Immune Epitope Database [34]. Fig 2A shows that IFN-γ responses to FYIQMLTEL could be detected in spleen cells of the positive control group that was vaccinated i.n. with a low virus dose. Furthermore, responses were detected in 25 SLP-vaccinated mice (alone or in combination with the B epitopes), but not in B epitopes vaccinated mice. Fig 2B shows responses to FYIQMLTEL only in inguinal LNs of 25 SLP-vaccinated mice but not in the virus vaccinated mice. This is not surprising, since the inguinal LNs are not the draining LNs of the lung, to where the i.n. virus vaccination is targeted. To AYERMCNIL, only very low responses were observed in the LNs. These results show that the T cell vaccine is capable of inducing influenza-specific T cell responses.

### Effect of Vaccination on Virus Challenge-Induced Clinical Manifestations in Mice

**Fig 3:**
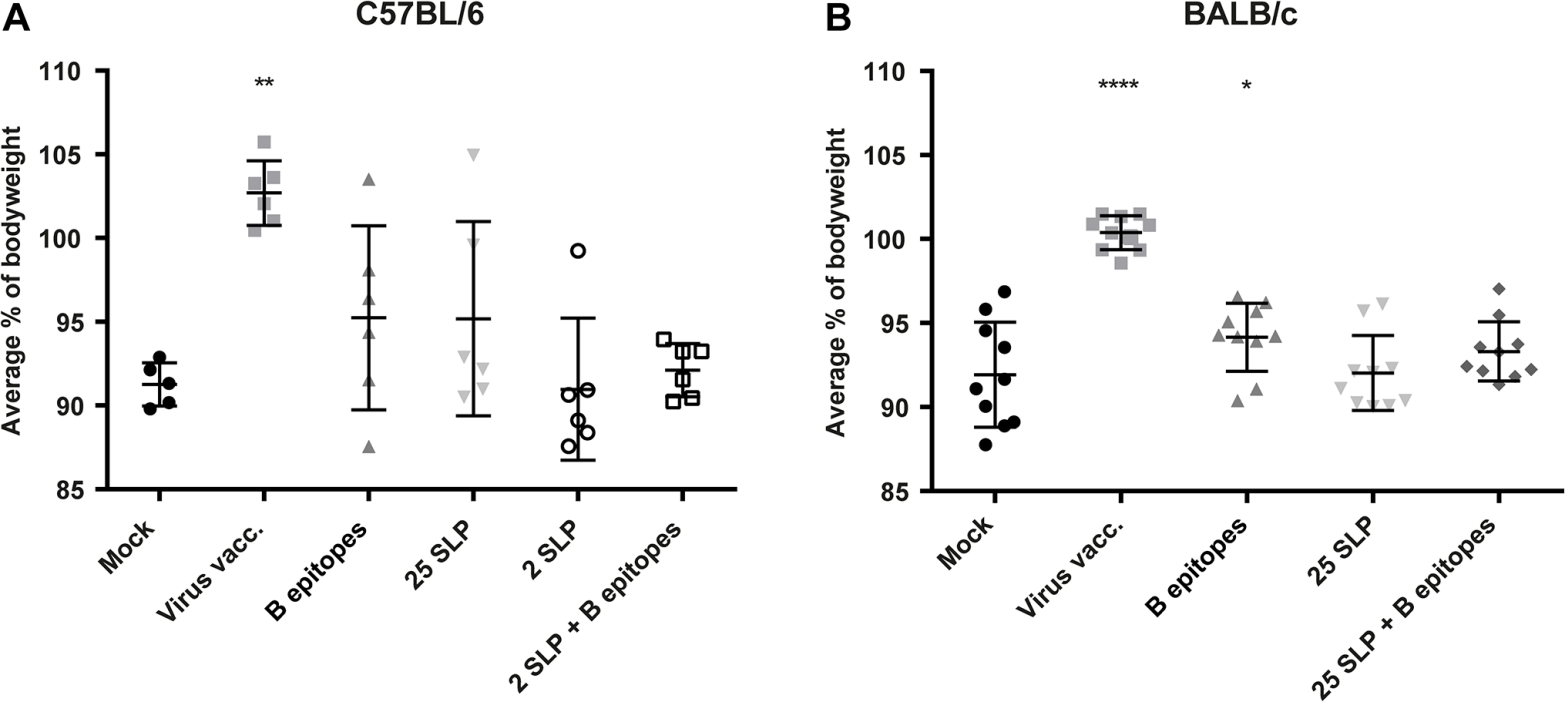
Bodyweight loss post challenge. C57BL/6 mice **(A)** and BALB/c mice **(B)** were challenged i.n. with 1*10^5^ TCID_50_ of HK-X31 virus and their bodyweight was recorded daily. Results are shown in average percentage of bodyweight relative to the bodyweight at the day of challenge. Data were log-transformed and analyzed for Gaussian distribution. C57BL/6 data were then analyzed with Mann-Whitney test and BALB/c data were analyzed with an unpaired t-test. *p = <0.05 **p = <0.01 ****p = <0.0001 compared to mock vaccinated animals.

Two weeks after booster vaccination, mice were challenged with a sub-lethal dose of HK-X31 virus and weighed daily for 14 days (S1 Fig). Fig 3 shows the average bodyweight during the challenge phase, calculated as the average percentage relative to the bodyweight at day of challenge. As a measure for recovery from disease, the average percentage bodyweight should be higher than in the negative control animals. However, all mice lost bodyweight, except the positive control group, which was completely protected. Mice vaccinated with 2 SLP lost bodyweight comparable to the mock vaccinated group, showing that such a minimal amount of peptides is not sufficient for protection from disease. C57BL/6 mice vaccinated with B epitopes and mice vaccinated with 25 SLP did show a slightly higher average bodyweight than mock-vaccinated mice although this is not a significant difference. In the experiment with BALB/c mice, the group size was enlarged to increase power. As expected, the positive control group did not lose any weight. Furthermore, recovery was significantly increased in mice vaccinated with B epitopes (p = <0.05). The other vaccine groups did not show a significant increase in weight recovery. Based on bodyweight, the vaccines show no major protection against loss of bodyweight, but do show some improved recovery.

### Effect of Vaccination on Virus Replication in Mice

**Fig 4:**
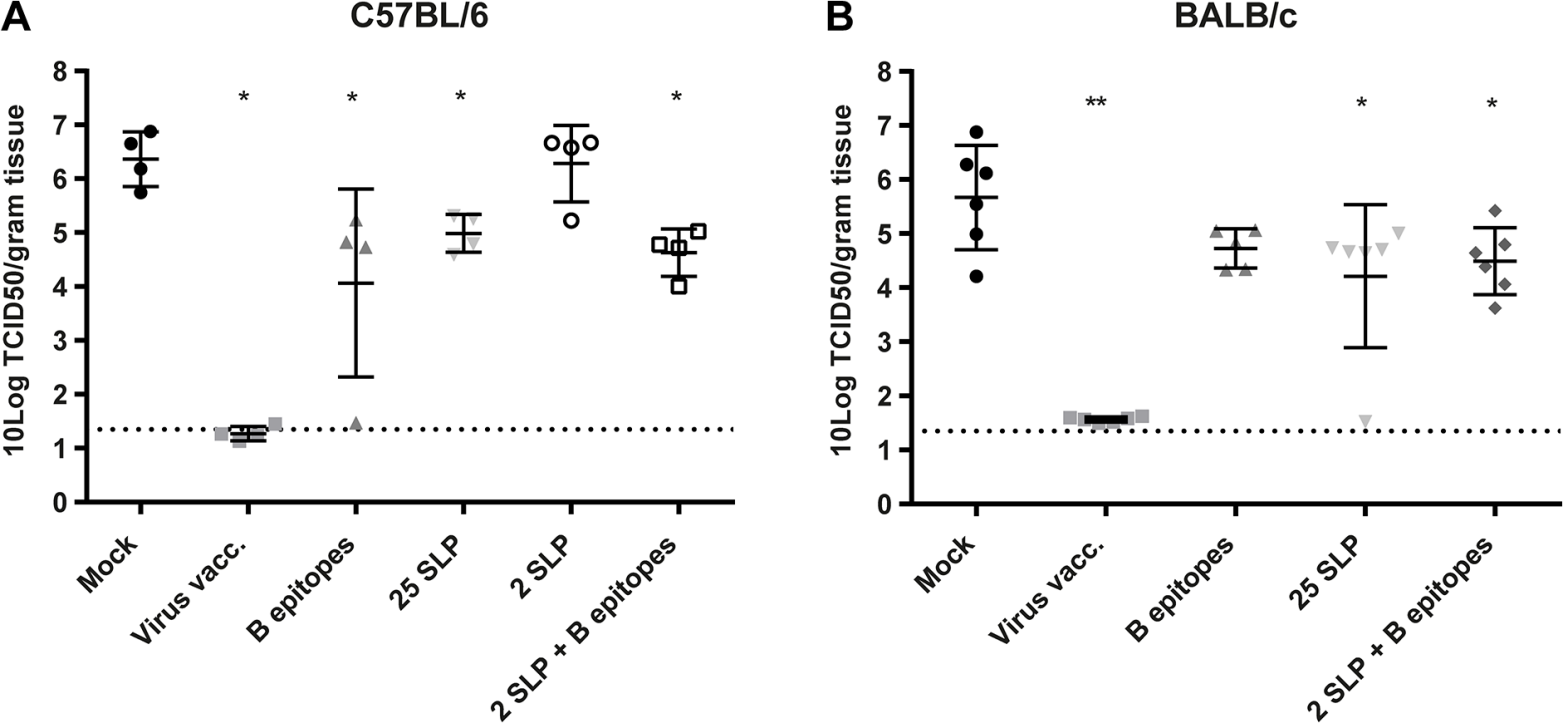
Reduced viral load in the lungs. Five days after challenge four (C57BL/6) and six (BALB/c) mice were sacrificed and virus titers in the lungs were determined by end point titration. Depicted are log transformed titers in the lungs for C57BL/6 mice **(A)** and BALB/c mice **(B)**. Data were analyzed by the Mann-Whitney test.*p = <0.05 **p = <0.01 compared to mock vaccinated mice.

As another parameter for vaccine efficacy, viral load in the lungs was analyzed five days after challenge using a TCID_50_ assay. A reduction in viral titers is an important measure of protection, since lower viral titers will lead to reduced spread of the virus. The positive control group (virus vaccination) was completely protected against virus replication in both C57BL/6 and BALB/c mice, as titers were below detection level. Fig 4 shows that vaccination with both 25 SLP and B epitopes achieved a 1.5–2 log reduction in viral load in the lungs of C57BL/6 mice and BALB/c mice. The combined vaccine achieved a comparable reduction as the B epitopes and 25 SLP alone. 2 SLP was the only vaccine that did not induce a reduction in viral load, indicating that the two long peptides alone are not sufficient for reducing viral load in the lungs. This shows that the peptide-based vaccines significantly reduced virus replication *in vivo*.

### Evaluation of Combined Vaccine in Ferrets

Our studies in mice showed that the B epitopes and 25 SLP induced antibody and T cell responses, respectively, and were capable of reducing viral load in the lungs. Next, we evaluated our vaccine concept in a ferret model. Ferrets respond clinically similar to humans to influenza infection and are therefore a commonly used follow up in pre-clinical vaccine development [35]. Both B and T cell responses are important in influenza infection and a universal vaccine should therefore target both the humoral and cellular immune response. For this reason, we only included the 25 SLP + B epitopes vaccine. Since route of immunization can also affect efficacy of vaccines, several routes of administration were evaluated in the ferret experiment. Pam3CysSK4 was included as an adjuvant in all vaccines and depending on the route of immunization, IFA was or was not added. IFA is a strong adjuvant for peptide vaccines that forms a depot at the site of injection and is not suitable for routes of immunization other than subcutaneously. Therefore, no IFA was included in the vaccines that were administered via i.m. and i.n. routes.

### Immunogenicity of B Epitopes in Ferrets

**Fig 5:**
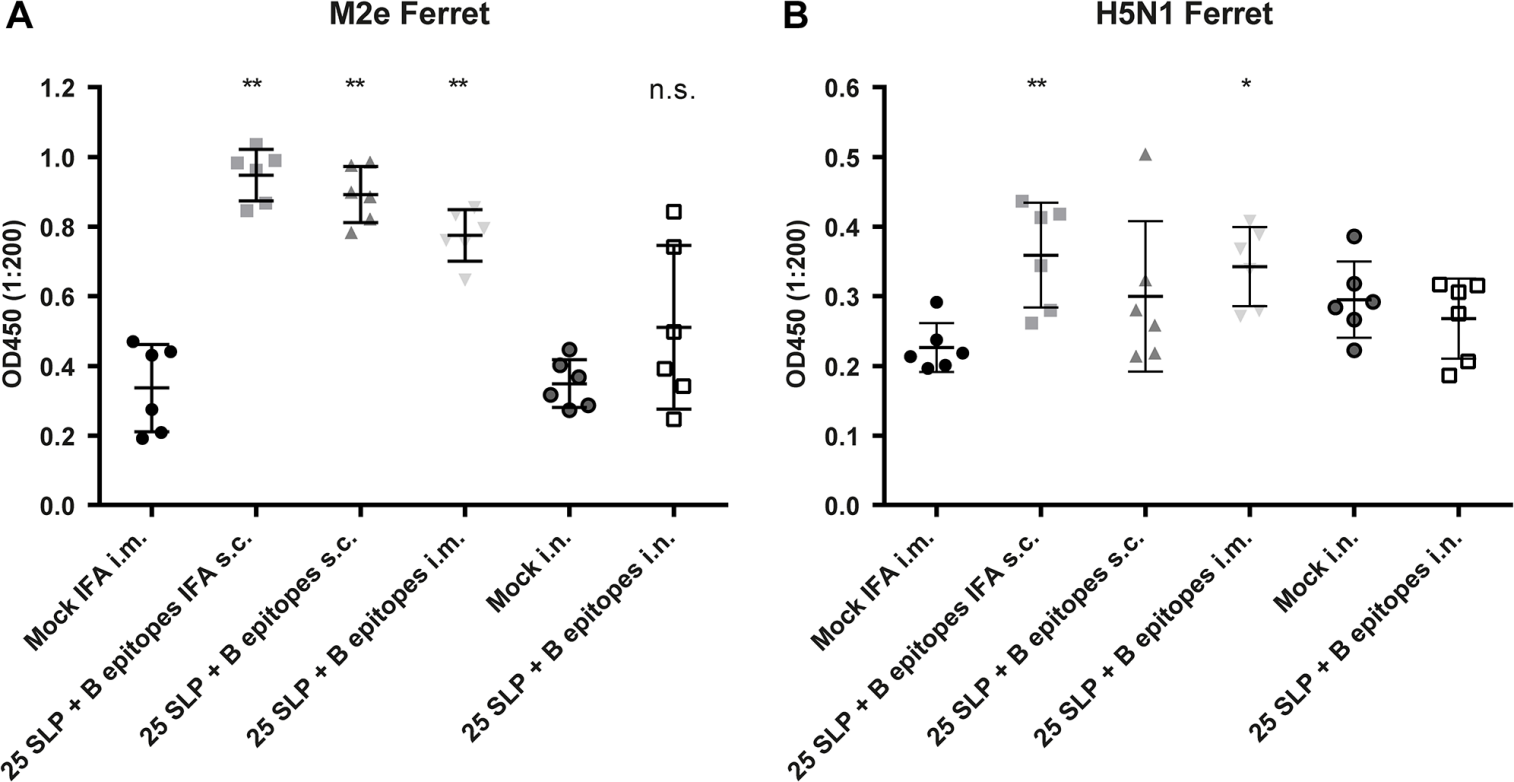
Immunogenicity of B epitopes in ferrets. IgG responses to the vaccines were measured in ferret sera collected two weeks after booster vaccination, by using M2e peptide **(A)** or whole inactivated influenza H5N1 virus vaccine **(B)** as ELISA antigens.

Sera collected two weeks after booster vaccination were analyzed for antibodies to HA2 and M2e peptide. No antibodies directed to HA2 peptide were detected in any of the vaccine groups. M2e-specific IgG antibody responses, on the other hand, were detected in sera of all ferrets vaccinated with 25 SLP + B epitopes i.m., s.c. and s.c. with IFA and in two ferrets that received the 25 SLP + B epitopes i.n. No antibodies were detected in the control groups (Fig 5A). To determine whether vaccine-induced antibodies recognize the epitopes in their natural conformation, an ELISA was performed with H5N1 WIV vaccine to enable analysis at BSL-2. The M2e-epitope of this vaccine is derived from the PR8 strain, which is 73.9% identical to the A/turkey/Turkey/1/2005 sequence. Of the groups that had detectable M2e antibodies, the ferrets that received 25 SLP + B epitopes IFA s.c. and 25 SLP + B epitopes i.m. also had detectable antibodies directed to WIV H5N1 (Fig 5B). This shows that the B epitopes are also immunogenic in the ferret model and that these antibodies are capable of recognizing the M2e epitopes even though sequences are not completely homologous.

### Effect of Vaccination on Virus Challenge-Induced Clinical Manifestations in Ferrets

Two weeks after booster vaccination, ferrets were i.t. challenged with H5N1 influenza virus. The administered challenge dose (1*10^5^ TCID_50_) was previously determined to be non-lethal in ferrets from a different provider, however a few ferrets in the current study succumbed or had to be sacrificed before scheduled termination. Two animals of the 25 SLP + B epitopes IFA s.c. group and one ferret of the 25 SLP + B epitopes s.c. group had to be terminated on the day prior to section, because they had reached the pre-determined clinical endpoint. One ferret of the mock i.n. group died in the night before section. However, these deviations from the original protocol only had a minor impact on the study. Four to five days after challenge, all ferrets became inactive and showed heavy breathing. No significant differences in bodyweight were observed in the peptide vaccine groups, all ferrets had lost approximately 15% of their bodyweight by day five (data not shown). Bodyweight of the positive control ferret previously vaccinated with virus by means of a low dose i.n. infection, did remain stable over the five-day challenge period. Since ferrets were sacrificed five days after challenge, which is the optimal day to measure virus titers, recovery in bodyweight could not be measured as this normally starts seven days after infection. During the five days after viral challenge, all ferrets developed fever, except for the positive control ferret. The ferrets vaccinated i.m. and s.c. with and without IFA also had a significantly lower temperature than the corresponding control (Table 2). Overall, the vaccine did not ease clinical symptoms of influenza virus infection, did not prevent initial body weight loss and only had a limited effect on preventing fever.

**Table 2:** Body temperature analysis of ferrets during viral challenge.

Group	AUC	SD	No. ferrets	Max ΔT (°C) #	SD	No. ferrets
Mock IFA i.m.	9.3	1.1	6	3.9	0.2	6
25 SLP + B epitopes IFA s.c.	7.4^*^	2.0	4	4	0.2	6
25 SLP + B epitopes s.c.	5.9^**^	0.7	5	3.6^*^	0.1	6
25 SLP + B epitopes i.m.	7.2^**^	1.9	6	3.7	0.1	6
Mock i.n.	8.3	0.9	5	3.8	0.2	6
25 SLP + B epitopes i.n.	9.3	2.5	5	3.8	0.3	5
Virus vacc.	0.6	n.a.	1	1.3	n.a.	1

# Max ΔT depicts the largest increase in temperature during the period after challenge.

Data were analyzed with a Mann-Whitney test.

* p = <0.05

** p = <0.01.

### Effect of Vaccination on Virus Replication in Ferrets

**Fig 6:**
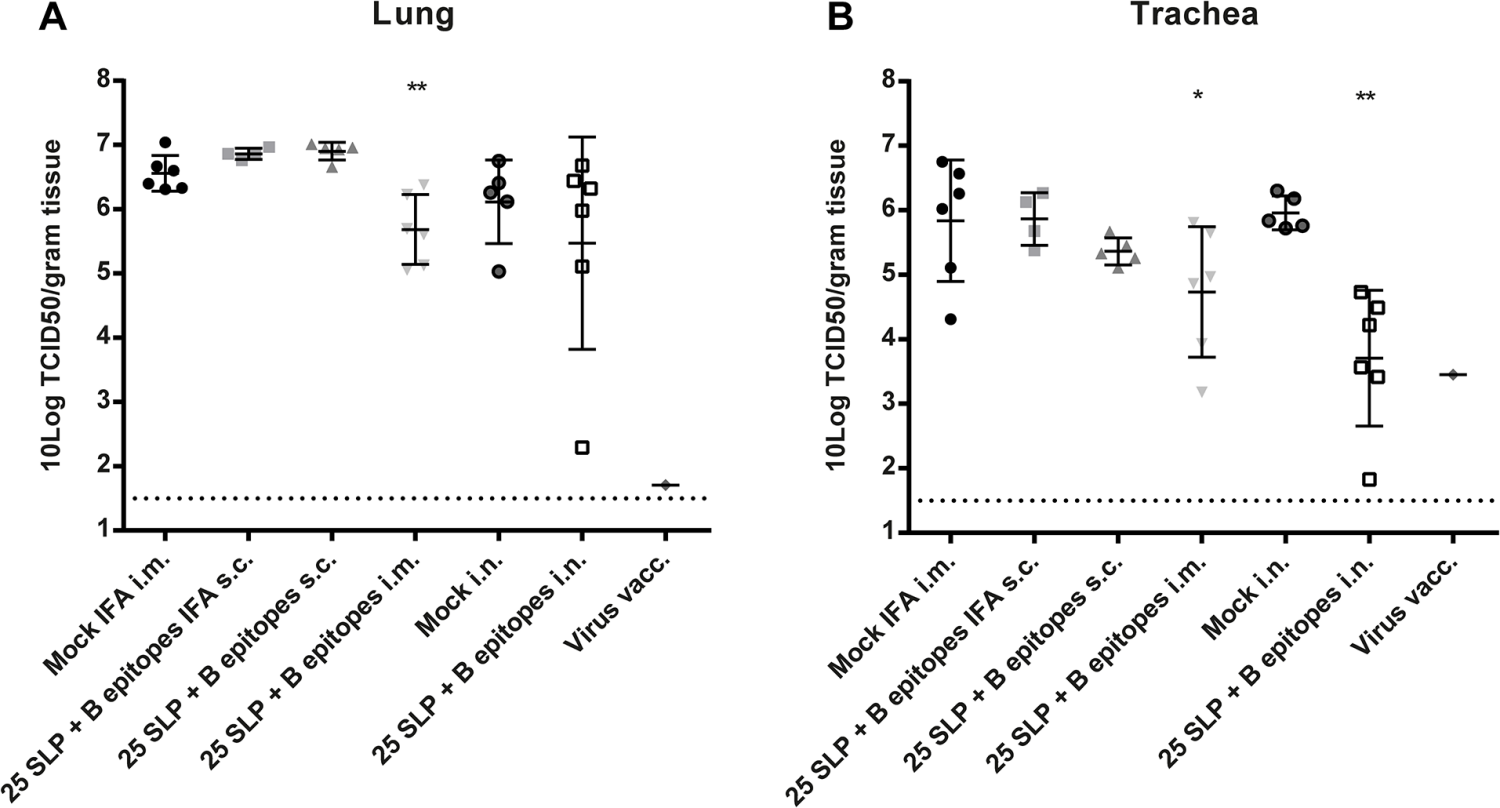
Virological analysis of lungs and trachea of ferrets. Five days after challenge ferrets were sacrificed and virus titers in the lungs and trachea were determined by end point titration. Depicted are log transformed titers in the lungs **(A)** and trachea **(B)**. Ferrets that had to be sacrificed or died prior to scheduled section were excluded from the analysis. Data were analyzed by the Mann-Whitney test.*p = <0.05 **p = <0.01 compared to mock vaccinated mice.

Fig 6A shows viral titers in the lungs. A significant difference is observed between the ferrets vaccinated i.m. with the 25 SLP + B epitopes and the corresponding negative control ferrets. Fig 6B shows viral load in the trachea, in which there is a significant difference observed between both the i.m. and i.n. 25 SLP + B epitopes vaccinated ferrets and their corresponding controls. The positive control ferret had no detectable virus titers in the lungs, but some virus remained detectable in the trachea. These results show that, depending on the route of immunization, the 25 SLP + B epitopes are capable of reducing virus load in both the lungs and the trachea of ferrets. Concluding, although vaccination of ferrets with the 25 SLP + B epitopes did not significantly ease symptoms of influenza virus challenge, the vaccine is capable of reducing virus titers in the lungs and trachea, which is especially of relevance from a transmission point of view.

## Discussion

Since the emergence of pH1N1 and spillover of potential pandemic H5N1 and H7N9 influenza viruses from the avian reservoir to humans, there has been a boost in research on vaccines that are capable of inducing cross-protection against multiple influenza A subtypes. In the past years, several strategies have been investigated in which conserved parts of influenza virus were targeted. In general, these strategies can be divided into antibody-inducing and T cell inducing-vaccines. Potential antibody-inducing universal vaccines often aim to induce antibodies directed to M2e or HA2 epitopes, which are two promising highly conserved antibody-inducing vaccine candidates [6, 36]. T cell responses, on the other hand, are often directed towards conserved parts of the internal proteins of the virus, such as NP, M1 and PB1 proteins [11, 12]. While antibodies can limit spread of the virus, T cells are responsible for elimination of the virus by killing virus-infected cells mainly by CD8^+^ T cells and CD4^+^ T cells have an important helper function in several arms of the immune response to viruses. Although T cell inducing vaccines cannot prevent infection, they might be advantageous by inducing rapid clearance of infection and limiting disease severity and virus transmission [37].

In this study, an antibody-inducing vaccine, a T cell-inducing vaccine, and a combined vaccine format were evaluated in C57BL/6 mice, BALB/c mice, and ferrets. The antibody-inducing vaccine consisted of M2e and HA2 fusion peptide. Both epitopes were conjugated to a highly conserved hemagglutinin-derived CD4 helper peptide or to the universal PADRE epitope, to increase immunogenicity of the peptides. In this study, we were indeed able to detect antibodies directed to M2e in C57BL/6 mice, BALB/c mice, and ferrets. Antibodies specific for the HA2 fusion peptide were, however, detected only in a few mice and ferrets.

The antigenic site on the HA2 fusion peptide was first described by Atassi *et al*, who showed that antibodies directed to the HA2 fusion peptide indeed could bind to synthetic HA2 fusion peptides [38]. A more recent study by Stanekova *et al* showed induction of antibodies after two immunizations of mice with HA2 fusion peptide conjugated to keyhole limpet hemocyanin (KLH). Following challenge with a lethal dose of homologous or heterologous influenza virus, these antibodies even induced effective cross-protection [39]. However, HA2 fusion peptide is known to be less immunogenic than M2e. [39, 40]. This might explain why, in our study, we were not able to detect antibodies directed towards HA2 fusion peptide. However, since we did find lower viral titers in the lungs of mice vaccinated with B epitopes alone, the inclusion of M2e peptide alone appears to be sufficient for reducing viral load in the lungs.

Because not only antibodies, but also T cells can play an important role in influenza infection, in our studies, also a T cell-inducing vaccine was included. The 25 SLP vaccine alone induced a 1.5–2 log reduction in viral titers in the lungs of mice, similar to that found with B epitopes alone. Furthermore, in BALB/c mice, vaccine-specific T cell responses were detected. There are several other articles describing peptide-vaccination strategies that successfully induce T cell responses and even achieve a certain amount of protection. For example, Matsui *et al* coupled CD8^+^ T cell-specific influenza epitopes to liposomes and were able to induce partial protection in mice. Tan *et al*. coupled several influenza epitopes to Pam2Cys, resulting in lipopeptides. Vaccination with these constructs reduced viral titers in the lungs of mice with 1–2 log compared to controls or led to increased survival in mice [41–45]. Some other strategies for inducing T cell responses to conserved peptides are reviewed by Pica *et al*. [46].

Although the peptide-vaccine strategies described above are promising, there are some limitations. The sequences used in these papers are indeed all highly conserved, but due to their length also restricted to a certain HLA molecule. Alexander *et al*. provided a solution by identifying several highly conserved influenza epitopes that can bind to more HLA types. These supertype epitopes could provide broad population coverage, and might therefore be interesting vaccine candidates [22]. We chose a different strategy by selecting longer peptides of minimally 26 amino acids to avoid the necessity for HLA typing. Next to CD8 epitopes, these long peptides also include possible CD4 epitopes. This strategy turned out be effective in patients with HPV-induced vulvar lesions [15]. In the past years, two Phase I clinical trials with Influenza peptide-based vaccines were published that implemented a similar strategy. In these studies, respectively four and six long peptides derived from highly conserved sequences, were selected and tested for their safety and immunogenicity. Both vaccines proved to be safe and immunogenic [47, 48]. Since our vaccine contains a larger amount of peptides, it has the potency to raise broader responses and cover even more HLA types.

By combining the B epitope and 25 SLP vaccine, we designed a vaccine capable of inducing both antibody and T cell responses against conserved regions and thus potentially providing broad protection. We evaluated this universal peptide influenza vaccine in C57BL/6 mice, BALB/c mice and ferrets. After viral challenge, the protective effects of the peptide vaccines based on clinical symptoms, such as fever and weight loss, were minimal. However, we did find a reduction of viral load in the respiratory tract of both ferrets and mice and although the vaccine did not completely protect the animals, vaccination did lead to some reduction in disease severity. Recently, Laidlaw *et al*. described that CTLs might act cooperatively with non-neutralizing antibodies. In this antibody dependent cell cytotoxicity (ADCC), killing of virus infected cells is mediated by complement or other cells of the innate immune system [8, 49, 50]. Although more research is needed, there are indications that the cooperation of CD8^+^ T cells, non-neutralizing antibodies, and alveolar macrophages can improve protection towards heterosubtypic viruses [51]. These findings promote development of a universal vaccine generating both antigen-specific CTLs and antibodies.

By optimizing the route of administration, the formulation and the adjuvants used in combination with the 25 SLP + B epitope, efficacy of the vaccine might be enhanced. Therefore, several routes were evaluated in the ferret model. Although ferrets vaccinated s.c. had M2e-specific antibodies in serum, these ferrets did not show a reduction in virus replication. Ferrets vaccinated i.m. and i.n., on the other hand, did show a reduction in viral titers. There are indications that the efficacy of CTL-inducing vaccines is increased by i.n. vaccination compared to s.c. vaccination, due to enhanced recruitment of CTLs to the lungs [42, 52]. Similar findings were observed for M2e, where only i.n. vaccination induced IgA-producing cells in the lungs of BALB/c mice [53].

Next to the induction of antibody- and T cell responses to conserved parts of the virus, there are other advantages to the use of peptides in a vaccine. In contrast to many of the currently implemented vaccines, peptides can be produced synthetically and are therefore often safer and easier to produce than vaccines containing biological agents. Furthermore, since there are practically no biological limitations, peptide vaccines can easily be produced in large amounts, which is convenient in case of a possible pandemic, when stock-piling is often needed [54].

At this point, a universal influenza vaccine is not expected to completely protect individuals. Reduced virus titers in the lungs will already lead to decreased spread of the virus, which might confine an outbreak to only a small area. Furthermore, disease severity in vaccinated individuals might be decreased and therefore lead to an increased survival rate. We show here that an easy-to-produce non-MHC biased peptide-based vaccine directed against conserved regions containing both B and T epitopes is able to reduce virus replication in lungs of mice and ferrets. After further development, this vaccine might potentially be used in a pandemic situation or supplementary to seasonal vaccines.

## Supporting Information

S1 Fig Bodyweight loss post challenge. C57BL/6 mice **(A)** and BALB/c mice **(B)** were challenged i.n. with 1*10^5^ TCID_50_ of HK-X31 virus and their bodyweight was recorded daily. Results are shown as average per group relative to the bodyweight at the day of challenge. Error bars depict SD per group.
(DOCX)

S1 Table List of known epitopes present in the synthetic long overlapping peptide vaccine. *nd = not determined.
(DOCX)
