# Supplementary material for: Synthetic Long Peptide Influenza Vaccine Containing Conserved T and B Cell Epitopes Reduces Viral Load in Lungs of Mice and Ferrets
Source: PLoS One. 2015 Jun 5;10(6):e0127969. doi: 10.1371/journal.pone.0127969 (PMC4457525; doi:10.1371/journal.pone.0127969)
Supplement: S1 Fig — C57BL/6 mice (A) and BALB/c mice (B) were challenged i.n. with 1*105 TCID50 of HK-X31 virus and their bodyweight was recorded daily. Results are shown as average per group relative to the bodyweight at the day of challenge. Error bars depict SD per group. (DOCX) [file pone.0127969.s001.docx]

**S1 Fig.**


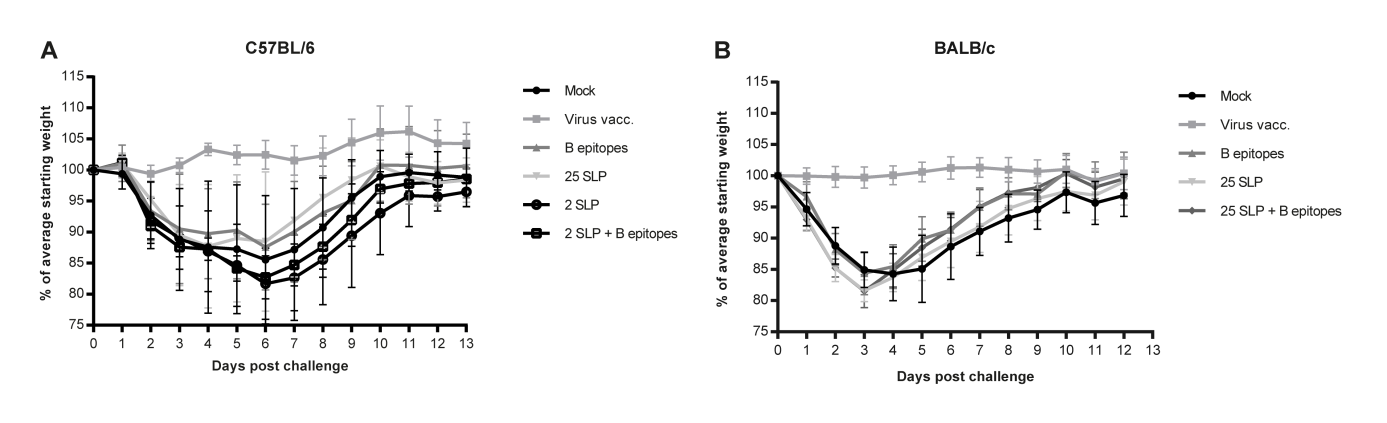


**Supplemental Figure S1:** **Bodyweight loss post challenge.** C57BL/6 mice **(A)** and BALB/c mice **(B)** were challenged i.n. with 1*10^5^ TCID_50_ of HK-X31 virus and their bodyweight was recorded daily. Results are shown as average per group relative to the bodyweight at the day of challenge. Error bars depict SD per group.

**B**

**A**
