# Supplementary material for: Synthetic Long Peptide Influenza Vaccine Containing Conserved T and B Cell Epitopes Reduces Viral Load in Lungs of Mice and Ferrets
Source: PLoS One. 2015 Jun 5;10(6):e0127969. doi: 10.1371/journal.pone.0127969 (PMC4457525; doi:10.1371/journal.pone.0127969)
Supplement: S1 Table — *nd = not determined. (DOCX) [file pone.0127969.s002.docx]

**S1 Table: List of known epitopes present in the synthetic long overlapping peptide vaccine.**

| **Peptide** | **Sequence** | **Specificity** | **Allele** | **Literature** |
| --- | --- | --- | --- | --- |
| NP_273-281_ | KSCLPACVY | CD8 | A1 | Alexander 2010 [[1](#_ENREF_1)] |
| NP_44-52_ | CTELKLSDY | CD8 | A1 | Wang 2007 [[2](#_ENREF_2)] |
| NP_329–339_ | QLVWMACHSAA | CD8 | A2 | Assarsson 2008 [[3](#_ENREF_3)] |
| NP_39-47_ | FYIQMCTEL | CD8 | A24 | Alexander 2010 [[1](#_ENREF_1)] |
| NP_67–76_ | RMVLSAFDER | CD8 | A3 | Assarsson 2008 [[3](#_ENREF_3)] |
| NP_383-391_ | SRYWAIRTR | CD8 | B27 | Wang 2007 [[2](#_ENREF_2)] |
| NP_17-25_ | GERQNATEI | CD8 | B44 | Alexander 2010 [[1](#_ENREF_1)] |
| NP_221–230_ | YERMCNILKG | CD8 | B44 | Assarsson 2008 [[3](#_ENREF_3)] |
| NP_338–346_ | FEDLRVSSF | CD8 | B44 | Assarsson 2008 [[3](#_ENREF_3)] |
| NP_199-207_ | RGINDRNFW | CD8 | B58 | Wang 2007 [[2](#_ENREF_2)] |
| NP_225-233_ | ILKGKFQTA | CD8 | B8 | Wang 2007 [[2](#_ENREF_2)] |
| NP_192–208_ | ELIRMIKRGINDRNFWR | CD4 | nd* | Lee 2008 [[4](#_ENREF_4)] |
| NP_264–281_ | LILRGSVAHKSCLPACVY | CD4 | nd* | Lee 2008 [[4](#_ENREF_4)] |
| NP_386–403_ | WAIRTRSGGNTNQQRASA | CD4 | nd* | Lee 2008 [[4](#_ENREF_4)] |
| NP_258–273_ | FLARSALILRGSVAHK | CD8 | nd* | Lee 2008 [[4](#_ENREF_4)] |
| NP_265-273_ | ILRGSVAHK | CD8 | nd* | Lee 2008 [[4](#_ENREF_4)] |
| NP_329–346_ | VWMACHSAAFEDLRVSSF | CD8 | nd* | Lee 2008 [[4](#_ENREF_4)] |
| NP_40–57_ | YIQMCTELKLSDYEGRLI | CD8 | nd* | Lee 2008 [[4](#_ENREF_4)] |
| PB1_30-38_ | YSHGTGTGY | CD8 | A1 | Alexander 2010 [[1](#_ENREF_1)] |
| PB1_489–497_ | TFEFTSFFY | CD8 | A1 | Assarsson 2008 [[3](#_ENREF_3)] |
| PB1_590-599_ | LVSDGGPNLY | CD8 | A1 | Alexander 2010 [[1](#_ENREF_1)] |
| PB1_591–599_ | VSDGGPNLY | CD8 | A1 | Assarsson 2008, Wang 2007 [[2](#_ENREF_2),[3](#_ENREF_3)] |
| PB1_407–415_ | MMMGMFNML | CD8 | A2 | Assarsson 2008 [[3](#_ENREF_3)] |
| PB1_412–421_ | FNMLSTVLGV | CD8 | A2 | Assarsson 2008 [[3](#_ENREF_3)] |
| PB1_413-421_ | NMLSTVLGV | CD8 | A2 | Alexander 2010 [[1](#_ENREF_1)] |
| PB1_501–509_ | FVANFSMEL | CD8 | A2 | Assarsson 2008 [[3](#_ENREF_3)] |
| PB1_505–514_ | FSMELPSFGV | CD8 | A2 | Assarsson 2008 [[3](#_ENREF_3)] |
| PB1_7–14_ | LLFLKVPA | CD8 | A2 | Assarsson 2008 [[3](#_ENREF_3)] |
| PB1_496-505_ | FYRYGFVANF | CD8 | A24 | Alexander 2010 [[1](#_ENREF_1)] |
| PB1_41-49_ | DTVNRTHQY | CD8 | A26 | Wang 2007 [[2](#_ENREF_2)] |
| PB1_488-497_ | GTFEFTSFFY | CD8 | A3 | Alexander 2010 [[1](#_ENREF_1)] |
| PB1_490–497_ | FEFTSFFY | CD8 | B44 | Assarsson 2008 [[3](#_ENREF_3)] |
| PB1_509–517_ | LPSFGVSGI | CD8 | B7 | Assarsson 2008 [[3](#_ENREF_3)] |
| PB1_540-548_ | GPATAQMAL | CD8 | B7 | Wang 2007, Lee 2008 [[2](#_ENREF_2),[4](#_ENREF_4)] |
| PB1_402-419_ | SLSPGMMMGMFNMLSTVL | CD4 | nd* | Lee 2008 [[4](#_ENREF_4)] |
| PB1_21-38_ | TFPYTGDPPYSHGTGTGY | CD8 | nd* | Lee 2008 [[4](#_ENREF_4)] |
| PB1_1–15_ | MDVNPTLLFLKVPAQ | CD4 | DR | Assarsson 2008 [[3](#_ENREF_3)] |
| PB1_404–418_ | SPGMMMGMFNMLSTV | CD4 | DR | Assarsson 2008 [[3](#_ENREF_3)] |
| PB1_408–422_ | MMGMFNMLSTVLGVS | CD4 | DR | Assarsson 2008 [[3](#_ENREF_3)] |
| M1_52–61_ | ILSPLTKGIL | CD8 | A2 | Assarsson 2008 [[3](#_ENREF_3)] |
| M1_58–66_ | GILGFVFTL | CD8 | A2 | Alexander 2010, Assarsson 2008 [[1](#_ENREF_1),[3](#_ENREF_3)] |
| M1_60–67_ | ILGFVFTL | CD8 | A2 | Assarsson 2008 [[3](#_ENREF_3)] |
| M1_100–110_ | LYRKLKREITF | CD8 | A24 | Assarsson 2008 [[3](#_ENREF_3)] |
| M1_179–188_ | RMVLASTTAK | CD8 | A3 | Assarsson 2008 [[3](#_ENREF_3)] |
| M1_48–58_ | KTRPILSPLTK | CD8 | A3 | Assarsson 2008 [[3](#_ENREF_3)] |
| M1_173-181_ | IRHENRMVL | CD8 | B39 | Wang 2007 [[2](#_ENREF_2)] |
| M1_196-205_ | SEQAAEAMEV | CD8 | B44 | Alexander 2010 [[1](#_ENREF_1)] |
| M1_173–189_ | IRHENRMVLASTTAKAM | CD4 | nd* | Lee 2008 [[4](#_ENREF_4)] |
| M1_180–199_ | VLASTTAKAMEQMAGSSEQA | CD4 | nd* | Lee 2008 [[4](#_ENREF_4)] |
| M1_55–72_ | LTKGILGFVFTLTVPSER | CD4 | nd* | Lee 2008 [[4](#_ENREF_4)] |
| M1_63–80_ | VFTLTVPSERGLQRRRFV | CD4 | nd* | Lee 2008 [[4](#_ENREF_4)] |
| M1_71–88_ | ERGLQRRRFVQNALNGNG | CD4 | nd* | Lee 2008 [[4](#_ENREF_4)] |
| M1_33–49_ | AGKNTDLEALMEWLKTR | CD8 | nd* | Lee 2008 [[4](#_ENREF_4)] |
| M1_40–57_ | EALMEWLKTRPILSPLTK | CD8 | nd* | Lee 2008 [[4](#_ENREF_4)] |
| M1_179–193_ | RMVLASTTAKAMEQM | CD4 | DR | Assarsson 2008 [[3](#_ENREF_3)] |
| M1_58–72_ | KGILGFVFTLTVPSE | CD4 | DR | Assarsson 2008 [[3](#_ENREF_3)] |

*nd = not determined

1. Alexander J, Bilsel P, del Guercio MF, Marinkovic-Petrovic A, Southwood S, et al. (2010) Identification of broad binding class I HLA supertype epitopes to provide universal coverage of influenza A virus. Hum Immunol 71: 468-474.

2. Wang M, Lamberth K, Harndahl M, Roder G, Stryhn A, et al. (2007) CTL epitopes for influenza A including the H5N1 bird flu; genome-, pathogen-, and HLA-wide screening. Vaccine 25: 2823-2831.

3. Assarsson E, Bui HH, Sidney J, Zhang Q, Glenn J, et al. (2008) Immunomic analysis of the repertoire of T-cell specificities for influenza A virus in humans. J Virol 82: 12241-12251.

4. Lee LY, Ha do LA, Simmons C, de Jong MD, Chau NV, et al. (2008) Memory T cells established by seasonal human influenza A infection cross-react with avian influenza A (H5N1) in healthy individuals. J Clin Invest 118: 3478-3490.

**Supplemental Figure**


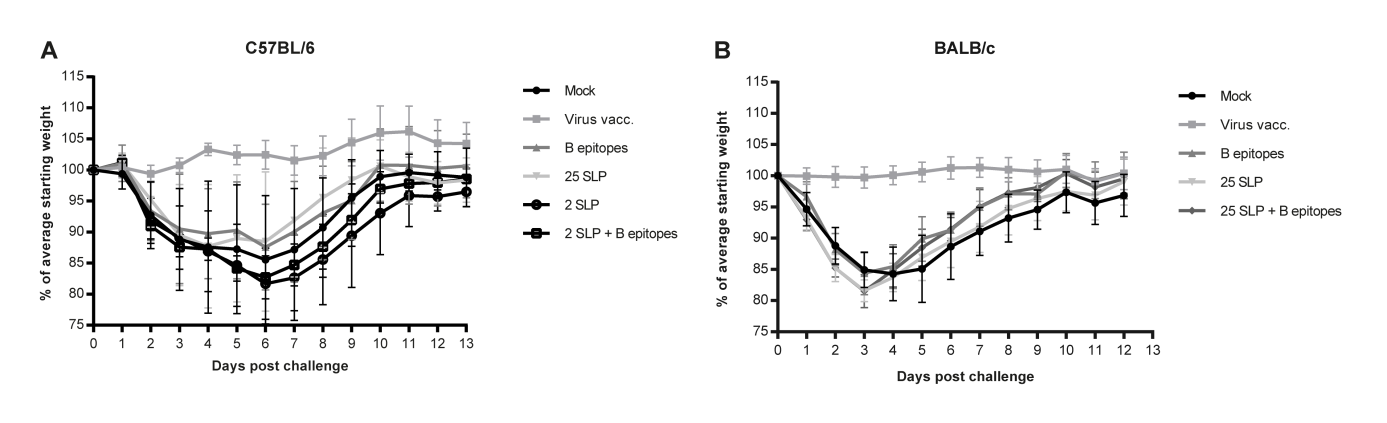


**Supplemental Figure S1:** *Bodyweight loss post challenge.* C57BL/6 mice **(A)** and BALB/c mice **(B)** were challenged i.n. with 1*10^5^ TCID_50_ of HK-X31 virus and their bodyweight was recorded daily. Results are shown as average per group relative to the bodyweight at the day of challenge. Error bars depict SD per group.

**B**

**A**
